# Pre-operative Factors Predicting Mortality in Six Months and Functional Recovery in Elderly Patients with Hip Fractures

**DOI:** 10.5704/MOJ.2303.002

**Published:** 2023-03

**Authors:** NH Nam, ND Minh, TX Hai, CT Sinh, CB Loi, LT Anh

**Affiliations:** 1 Department of Orthopaedics, Nghe An Orthopaedic and Trauma Hospital, Nghe An, Vietnam; 2 Department of Orthopaedics, National Hospital of Acupuncture, Ha Noi, Vietnam; 3 Department of Pediatrics, Nghe An Obstetrics and Pediatrics Hospital, Nghe An, Vietnam; 4 Department of Orthopaedics Vinh Medical University, Nghe An, Vietnam; 5 Department of Parasitology, National Institute of Malariology, Parasitology and Entomology; 6 Department of Parasitology, Vietnam Military Medical University, Hanoi, Vietnam

**Keywords:** hip fracture, mortality, functional recovery, harris hip score, Vietnam

## Abstract

**Introduction:**

This study aimed to determine on-admission and perioperative factors predicting six-month mortality and functional recovery in Vietnamese patients with hip fracture.

**Materials and methods:**

Between April 2020 and July 2021, 118 patients participated in this prospective study. Patients’ data were collected from medical records. Harris hip score (HHS) was used to evaluate the functional recovery six months after fractures. The obtained data were analysed using a univariate and multivariate model.

**Results:**

The mean age of the participants was 79.5±9.4 years and 68.6% of the patients were female. The six-month mortality rate was 5.9% and independently associated with age (odds ratio (OR): 3.512, 95% confidence interval (CI) 1.538 – 8.019; P<0.001, patients aged >80 years vs those aged ≤80 years) and hypoproteinemia (OR: 2.859, 95% CI: 1.001 – 8.166, P=0.049). Among 111 survivors there were 66 (59.5%) of patients with a good functional recovery. Patients aged >80 years had a higher risk of poor functional outcome (OR: 3.167, 95% CI: 1.386 – 7.235, P: 0.006) compared to those aged ≤ 80 years. No significant correlations between other clinical (gender, body mass index, comorbidities, type of fractures or surgery, time until surgery) or laboratory parameters (anaemia, hyperglycemia, marked elevation of C reactive protein level, electrolyte abnormalities, elevated urea) and mortality or functional outcome were found.

**Conclusion:**

Advanced age is the most important factor affecting both mortality and functional outcome while hypoproteinemia is associated with a higher risk of mortality in elderly patients with hip fractures.

## Introduction

Hip fractures are breaks of the upper portion of the femur (thigh bone) which are estimated to affect about 18% of women and 6% of men worldwide^1^. This devastating injury commonly occurs in the elderly and put patients at an increased risk of adverse outcomes, such as early death or functional decline. The first six months post-fracture is the most critical time as the risk of mortality is the highest and functional recovery occurs mostly between three and six months after fractures^2-5^. Thus, understanding the factors predicting six-month mortality and the functional recovery has become a central issue and received considerable attention. However, most data in this field have been from Western developed nations, and little is known about factors related to short-term mortality and functional recovery of hip fracture patients in Asian countries^6^.

Vietnam is a Southeast Asia country with a rapidly ageing population^7^. It is predicted that more than one-quarter of the total population aged over 60 years old by 20497. As a result of the rapid increase in the elderly population, the number of hip fracture patients is also expected to increase. Compared to hip fracture patients from developed nation, Vietnamese patients have different demographic or clinical characteristics^8-10^. Consequently, the factors correlated to mortality or functional outcome among hip fracture patients may be different from those in developed countries. For this reason, the data from Vietnam are useful for clinical implementation and further scientific understanding as well. As far as we know, no previous research has directly investigated this topic in Vietnam^10,11^.

The objectives of this research were to determine whether any on-admission and perioperative factors, especially manageable ones, were associated with six-month mortality and functional outcome in a Vietnamese cohort.

## Materials and Methods

This study is part of thesis work for the fulfilment of Doctor of Philosophy in Health Studies at the National Institute of Malariology, Parasitology, and Entomology (NIMPE) of Vietnam. The research received ethical clearance from the NIMPE review board on February 24, 2020 (182/QD-VSR). Written informed consent was obtained from all patients or their legal representatives after receiving an explanation of the study.

This prospective survey was conducted in Nghe An Orthopaedic and Trauma Hospital, Vietnam, between April 2020 and July 2021. Participants were all patients with fractures of the proximal end and agreed to participate in this study. Patients with undisplaced fractures or multisystem trauma were excluded. Participants’ data were collected from the medical records using a standardised case report form. The admission data in this work consisted of demographic variables (gender, age, body mass index (BMI), clinical variables (medical history, comorbidity, type, cause of fractures), laboratory findings (results of blood biochemical tests or cell counts, imaging analysis, bone mineral density (BMD) of the neck of the contralateral femur). Perioperative data included type of surgery (internal fixation or hip arthroplasty (HA), waiting time from the admission to surgery (WTS). The outcome variables were mortality and functional recovery six months post-fractures.

A conventional protocol was applied to all the patients. All comorbidities were treated prior to surgery. Patients with Garden I or II fractures received fixation while partial hip replacements were indicated for fractures of Garden types III and IV. All patients were instructed by rehabilitation specialists to perform in-hospital and at-home functional rehabilitation. The subjects were followed by telephone to determine survival status. The functional outcome was evaluated using Harris hip score (HHS) at six months after the injury^12^.

The Asian criteria for BMI (Kg/m^2^) were used to categorise patients (underweight (<18.5), normal weight (18.5 – 23), overweight and obesity (≥23)^9^. Anaemia was defined as a haemoglobin level below 8.1mmol/L [13 g/dL] in men and below 7.5mmol/L [12g/dL] in women^13^. The cutoff value for hyperglycemia was random plasma glucose ≥200mg/dL (11.1mmol/L) or fasting plasma glucose ≥126mg/dL (7.0mmol/L)^14^. Patients having systolic blood pressure (BP) ≥140mm Hg and/or diastolic BP ≥90mm were considered to have raised BP^15^. The normal ranges of laboratory parameters were as follow: protein 6–8g/dL, albumin 3.5–5.0g/dL, sodium 135–145mmol/L, potassium 3.6–5.0mmol/L, chloride 95–107mmol/L, calcium (total) 2.1–2.6mmol/L, urea 2.5–6.6mmol/L, creatinine 60–120μmol/L^16^. The C Reactive Protein (CRP) levels were categorised as normal or moderate elevation (≤10.0mg/dL) and marked elevation (>10.0mg/dL)^17^. Bone mineral density were categorised based on the T-scores as follows: normal (≥-1.0), low bone mass (osteopenia) (-1 to -2.5), or osteoporosis (≤-2.5)^18^. The cut point for "early" and "delayed" was the operation performed within 48 or after 48 hours of hospital admission, respectively^19^. Patients with an HHS≤80 were considered to have fair recovery while those with HHS>80 were the good^12^.

Categorical variables were reported by frequencies and percentages while continuous covariates were expressed as means and standard deviations (SD). Pearson's chi-square tests and, when appropriate, Fisher exact tests were used for univariate analysis. Independent variables that had an association (e.g., p<0.05) on univariate analysis were entered into a multivariable linear regression model to analyse the effect of these variables on mortality and functional outcome. A P value less than 0.05 was considered statistically significant for all analyses. Statistical analysis was performed using SPSS statistical software version 16.0.

## Results

There were 118 patients aged between 61 and 101 years (mean of 79.5) included in the study. Among them 66.9% had at least one comorbidity. Hypertension (50.8%) and diabetes mellitus (DM; 40.7%) were the most frequent comorbidities. Coronary artery disease (10.2%), pulmonary disease (7.6%), gastrointestinal disease (4.2%), and neurologic disorders (2.5%) were the other comorbidities reported from our sample. The most frequent cause of the injury was a low-impact fall (95.8%). The most abnormalities in laboratory parameters were the elevation of C-reactive protein (CRP) level, followed by decreased mineral density, anaemia, and hypoalbuminemia. The most common imbalance in electrolyte disorders was decreased sodium levels. Regarding the operation, 67.0% of the patients underwent partial HA, and fixation was indicated for 30.5% of patients.

The minority of patients were female (68.6%), with female: male ratio of 3:1. Compared to males, females had a lower value of BMI, and a higher rate of DM. Females were more likely to suffer from a fracture due to fall, intertrochanteric fracture, and had partial HA. Women had a significantly lower T-score and a higher rate of osteoporosis compared to men. All other findings were nearly comparable in both genders. The 6-month mortality rate was 5.9%. Among 115 survivors there were 66 (59.5%) patients with a good functional recovery (Table I).

**Table I:** Clinical and laboratory data of the study population (n=118)

Parameter	Value (n, %, CI95% or mean±SD)
Age (years)	79.5±9.4
≤ 80	63 (53.4, 44.3 – 62.5)
> 80	55 (46.6 (37.5 – 55.7)
Gender	
Male	37 (31.4, 21.9 – 40.1)
Female	81 (68.6, 57.7 – 79.5)
BMI	19.6±1.8
Underweight	31 (26.3, 17.3 – 35.3)
Normal weight	82 (69.5, 60.5 – 78.5)
Overweight and obese	5 (4.2, 0.5 – 7.9)
Comorbidity	79 (66.9, 58.3 – 75.6)
Hypertension	60 (50.8, 39.8 – 61.8))
Diabetes mellitus	48 (40.7, 29.7 – 51.6)
Cause of fracture	
Fall	113 (95.8, 86.8 – 104.8)
Traffic accident	5 (4.2, 0.6 – 7.9)
Type of fracture	
Femoral neck fracture	45 (38.1, 29.1 – 47.1)
Intertrochanteric fracture	73 (61.9, 52.9 – 70.9)
Laboratory data	
Anemia	96 (81.4, 72.4 – 90.4)
Low protein	24 (20.3, 11.3 – 29.3)
Low albumin	78 (66.1, 57.1 – 75.1)
Normal	20 (16.9, 7.9 – 25.9)
Osteopenic	54 (45.8, 36.8 – 54.8)
Osteoporotic	44 (37.3, 28.3 – 46.3)
Marked elevation of CRP level	83 (70.3, 61.3 – 79.3)
Elevated ure	39 (33.1, 24.1 – 42.1)
Elevated creatinin	1 (0.8, -0.8 – 2.5)
Decreased sodium level	57 (48.3, 39.3 – 57.3)
Decreased calcium level	32 (27.1, 18.1 – 36.1)
Decreased potassium level	22 (18.6, 9.6 – 27.6)
Elevated chloride level	18 (15.3, 6.3 – 24.3)
Abnormal electrolytes	82 (69.5, 60.5 – 78.5)
WTS (hours)	52.1±33.9
≤48 hours	53 (45.8, 36.6 – 54.9)
>48 hours	65 (54.2, 45.1 – 63.4)
Type of treatment	
No	3 (2.5)
Fixation	36 (30.5, 21.5 – 39.5)
Partial HA	79 (67.0, 57.9 – 76.0)
Mortality	7 (5.9, 1.6 – 10.3)
Good function recovery†	66 (55.9, 46.8 – 65.2)

† for 111 survival patiens

The univariate analysis showed that advanced age, hypoproteinemia, and osteoporosis were significantly correlated with mortality. Results of multivariate analysis indicate that advanced age (odds ratio (OR): 3.512, 95% confidence interval 1.538 – 8.019; P<0.001) and hypoproteinemia (OR: 2.859, 95% CI=1.001 – 8.166, P=0.049) were factors significantly predicting 6-month mortality. The mean age was 18.7% higher in the 7 deceased patients than in the 111 survival subjects (93.3±2.9 vs 78.6±8.9 years, P<0.001). No significant correlations between other clinical or laboratory parameters and mortality were found (Table II).

**Table II:** Predictor variables of mortality six months after fractures (n=118)

	Univariate analysis		Multivariate analysis	
	OR (CI 95%)	P	OR (CI 95%)	P
Age group*	1.083 (1.002 – 1.172)	0.039†	3.512 (1.538 – 8.019)	0.003†
Gender	1.310 (0.600 – 2.860)	0.552		
BMI‡	0.742 (0.321 – 1.713)	0.533		
Hypertension	0.941 (0.455 – 1.974)	1.000		
Type of fracture	1.129 (0.533 – 2.390)	0.849		
BMD§	2.290 (1.070 – 4.905)	0.036†	1.688 (0.728 – 8.816)	0.223
Diabete mellitus	0.978 (0.467 – 2.051)	1.000		
Hypoproteinemia	4.094 (1.545 – 10. 848)	0.005†	2.859 (1.001 – 8.166)	0.049†
Anaemia	0.933 (0.368 – 2.368)	1.000		
CRP level||	1.783 (0.785 – 4.048)	0.233		
Hyponatremia	1.489 (0.717 – 3.090)	0.354		
Hypokalemia	0.408 (0.147 – 1.131)	0.098		
Hypocalcemia	1.169 (0.517 – 2.640)	0.385		
Elevated chloride	1.018 (0.371 – 2.796)	1.000		
Elevated urea	1.546 (0.714 – 3.346)	0.326		
Electrolyte abnormality	0.978 (0.444 – 2.153)	1.000		
Waiting time¶	0.588 (0.281- 1.231)	0.194		
Type of surgery#	1.029 (0.475 – 2.230)	1.000		

Notes - *Age (≤80 vs >80 years), † significant P<0.05, ‡ underweight patients compared to normal and overweight ones, § comparing between patients with osteoporosis and those with normal or low bone mass (osteopenia), || marked elevation and normal or moderate elevation (≤10 vs >10μmol/L), ¶ ≤48 vs >48 hours, #fixation vs partial HA.

There was a significant association between age and hypoproteinemia with poor outcome of function upon univariable analysis (p<0.05). However, age was the only factor that significantly affected the functional recovery on multivariate analysis. The mean age of patients with good functional outcomes was smaller compared to those with fair outcomes (76.4±8.2 vs 82.8±9.3 years, P<0.001). The average HHS for patients ≤80 years was significantly higher than that among those >80 years (84.2±8.0 vs 75.5±22.2 HHS, P<0.01). No significant association was found between other clinical or laboratory data and functional outcomes (Table III).

**Table III:** Factors predicint functional recovery six months after fractures (n=111)

	Univariate analysis		Multivariate analysis	
	OR (CI 95%)	P	OR (CI 95%)	P
Age group*	3.788 (1.703 – 8.424)	0.001†	3.167 (1.386 – 7.235)	0.006†
Gender	1.365 (0.607 – 3.069)	0.534		
BMI‡	0.707 (0.293 – 1.707)	0.513		
Hypertension	1.176 (0.550 – 2.518)	0.703		
Type of fracture	1.439 (0.646 – 3.207)	0.424		
BMD§	1.979 (0.895 – 4.376)	0.107		
Comorbitidy	0.871 (0.381 – 1.990)	0.835		
Diabete mellitus	0.963 (0.445 – 2.085)	1.000		
Hypoproteinemia	3.806 (1.392 – 10.410)	0.012†	2.762 (0.960 – 7.947)	0.060
Hypoalbuminemia	1.501 (0.665 – 3.388)	0.416		
Anaemia	0.889 (0.340 – 2.325)	0.810		
CRP level||	1.848 (0.797 – 4.283)	0.215		
Hyponatremia	1.458 (0.681 – 3.121)	0.342		
Hypokalemia	0.481 (0.172 – 1.343)	0.225		
Hypocalcemia	1.171 (0.501 – 2.735)	0.828		
Elevated chloride	0.862 (0.289 – 2.565)	1.000		
Elevated urea	1.502 (0.672 – 3.357)	0.409		
Electrolyte abnormality	1.070 (0.466 – 2.458)	1.000		
Waiting time¶	0.627 (0.291 – 1.351)	0.251		
Type of surgery#	1.107 (0.491 – 2.495)	0.839		

Notes - *, †, ‡, §, ||, ¶, #: symbols as in Table II. The analysis was done for 111 survival patients only.

## Discussion

The first aim of the current study is to find out predictors of six-month mortality in hip fracture patients. Our results demonstrated that advanced age and hypoproteinemia were the independent factors predicting six-month mortality. The list of factors affecting early mortality after hip fracture is very extensive, however, the influence of these factors are variable and, sometimes, conflicting. A number of studies have showed that demographic characteristics such as age^4,20,25^, gender^20-22,24,25^, BMI^26^ are predictors of early mortality in patients with hip fractures. Clinical findings like comorbidities^20,23-25,27-29^, type of fracture^20,28-30^, type of surgery^20,24^, and time until surgery^23,24^ may also have an effect on early mortality after hip fractures. Similarly, laboratory anormalities including low level of hemoglobin^21,31^, albumin^26,31-34^, high levels of ure^35^, creatinine^21,31,35,36^, CRP^26^, sodium^35^, potassium^35^ have been found to associated with increased mortality. On the other hand, other reports have found no correlation between gender^23,28,29,36^, comorbidities^26,33^, type of fracture^37^, pre-fracture function^29^ or laboratory disorders^33,34,36,38^ and the risk of early mortality among hip fracture patients. The findings of our study reconfirm that advanced age is one of the factors that predict early mortality in elderly patients with hip fractures. Such finding is validated by results from a recent review which showed that age was the only factor influencing mortality risk in all (20/20) studies under reviewed^39^. The association between hypoproteinemia and increased risk of six-month mortality in our patients is supported by several earlier observations^31-34^. This association may have a significant clinical implication because hypoproteinemia is a modifiable factor while advanced age is an unmodifiable factor. Measures to control hypoproteinemia such as nutritional supplementation may serve as a key target for peri- and postoperative management to increase survival opportunities for hip fracture patients^40^. It is interesting to note that the prevalence of overweight in our sample was low (4.2%) compared to reports in developed countries (about 20%)^41^. However, the difference in bodyweight had no effect on the outcome of the patients which is consistent with those from other studies^33^.

The second aim of the current study is to identify factors that may predict functional outcomes after hip fractures as mortality and functional status ideally should be considered both together^42^. Our result shows that advanced age is the only factor that significantly affects the functional recovery of patients with hip fractures (Table III). Many attempts have been made to identify factors predicting functional outcomes, but the results are inconsistent^6,23,30,38,43-53^. For instance, comorbidity has been found to be a predictor of functional outcome in the studies done by Fox *et al* (1999)^30^, Shyu *et al* (2004)^6^. On the contrary, Maroffoli *et al* (1992) found that functional scores six months post-fracture were not significantly different between patients with more than two comorbidities and those having one or no comorbidity^45^. Slaven EJ (2012) demonstrated that gender was the only individual variable that predicted functional outcomes at six months^54^, but many other researchers have failed to address the difference in functional outcomes between the two genders^23,45,46^. The finding of age as the only independent predictor of functional outcome among patients with hip fracture in our study was corroborated by Cornwall R *et al* (2004)^37^.

One concern about the findings of our study was that is a small sample and a single-centre study. In addition, the hospital-related factors (such as the type, volume of the hospital) have not been investigated in the current study. Nevertheless, our results can be generalised to elderly patients with hip fractures in other level II centres. The setting in our study is a provincial hospital for trauma and orthopaedics which gives services similar to many level II centres around the world. Our study population is nearly comparable to hip fracture patients worldwide regarding demographic and clinical characteristics (Table I). These similarities suggest that independent factors affecting six-month mortality or functional recovery in our sample could be applicable to other patients suffering from this injury.

## Conclusion

This study provided preliminary knowledge concerning pre-and perioperative predictors for mortality and functional outcome following hip fractures in Vietnam. The present study has identified advanced age as an independent predictor of mortality and poor functional outcome as well as figured out the association between hypoproteinemia and mortality after hip fracture. We believe uncovering these data is useful both for providing a precise prognosis and a more adequate intervention strategy (e.g., nutritional supplementation for patients with hypoproteinemia) for hip fracture patients.
